# Resting state and personality component (BIS/BAS) predict the brain activity (EEG and fNIRS measure) in response to emotional cues

**DOI:** 10.1002/brb3.686

**Published:** 2017-04-05

**Authors:** Michela Balconi, Maria E. Vanutelli, Elisabetta Grippa

**Affiliations:** ^1^ Research Unit in Affective and Social NeuroscienceCatholic University of the Sacred HeartMilanItaly; ^2^ Department of PsychologyCatholic University of the Sacred HeartMilanItaly

**Keywords:** behavioral inhibition system/behavioral activation system, electroencephalography, emotion, functional near‐infrared spectroscopy, lateralization, resting state

## Abstract

**Introduction:**

The present study explored the role of resting state and personality component (BIS/BAS measure) on prefrontal cortical responsiveness to emotional cues. Indeed, we supposed that lateralized resting activity (right vs. left) and approach (BAS) versus avoidance (BIS) attitude may explain the successive emotional processing within the prefrontal cortex (PFC) based on the stimulus valence (positive and negative emotional cues).

**Methods:**

Hemodynamic (functional near‐infrared spectroscopy, fNIRS) and electroencephalographic (EEG) measures were considered. The resting and experimental brain activity were registered when subjects (*N* = 21) viewed emotional positive versus negative stimuli (International Affective Picture System, IAPS). LIR_eeg_ and LIR_nirs_ (lateralized Index Response) during resting state, and LI_eeg_ and LI_nirs_ during emotional processing were acquired.

**Results:**

A set of regression analyses was applied to the multiple measures. The predictive effect of resting activity and approach/avoidance dichotomy were elucidated. Indeed, more left/right resting activity (for both LIR_eeg_ and LIR_nirs_) predicted the successive more brain left/right response (LI_eeg_ and LI_nirs_) to emotional cues. Second, significant effects were revealed as a function of valence (increased right response to negative stimuli; increased left response to positive stimuli) during emotion processing. Third, higher BAS values explained an increased left cortical activity in resting state and in experimental condition for positive cues. In contrast, higher BIS values marked an increased right activity in resting state and in experimental condition in response to negative cues.

**Conclusion:**

The significance of trait component for both resting and emotional cue processing was discussed at light of the present results.

## 1 Introduction

Research on frontal brain asymmetry and emotion is one of the most relevant domains in the neuroscience domain. The studies associated with frontal asymmetry, for instance, include child temperament (Fox, Henderson, Rubin, Calkins, & Schmidt, 2001), self‐report measures of affect and personality (Tomarken & Davidson, 1994), social anxiety (Schmidt, 1999), or social status (Tomarken, Dichter, Garber, & Simien, 2004). Two main models were adopted to describe subjective asymmetries in brain activity within the frontal areas, that is the dispositional model of affective style, which postulates that people possess a general tendency to respond predominantly with either an approach or a withdrawal behavior independently from the situational differences (Balconi & Mazza, 2010; Davidson, 1998); the valence model, which supposes that cortical differences between the two hemispheres are attributable to positive versus negative valence of emotional conditions (Everhart, Carpenter, Carmona, Ethridge, & Demaree, 2003; Russel, 2003; Silberman & Weingartner, 1986).

Based on the valence model, the right hemisphere is specialized for negative emotions and the left hemisphere for positive emotions. In general, this model was applied to expression and perception of emotions, as well as to emotional experience. Some interesting results were found in response to specific emotional conditions. In particular, sadness was positively correlated with more right alpha power and negatively with left alpha power, whereas happiness was mainly related to left‐side activation (Davidson & Fox, 1982).

In addition, the “dispositional” perspective considered some personality components, such as trait component, able to explain the inter‐subjective differences in frontal asymmetry for emotion processing. Based on the integrated and new approach–withdrawal model of emotion, emotional behavior should be related to a balance of activity in left and right frontal brain areas that can be described using an asymmetry measurement (Harmon‐Jones & Allen, 1997; Sutton & Davidson, 2000).

Specifically, resting frontal asymmetry measured by electroencephalography (EEG) was related to appetitive (approach‐related) and to aversive (withdrawal‐related) motivation: approach tendencies should be reflected in left‐frontal activity and heightened withdrawal tendencies in relative right‐frontal activity (Balconi & Lucchiari, 2005, 2007; Balconi & Pozzoli, 2003; Stewart, Coan, Towers, & Allen, 2014).

Indeed, much research has documented the trait properties of frontal EEG asymmetry, which predicts that more resting left active individuals are more responsive to positive stimuli (such as happiness) and higher in approach attitude (such as anger), whereas more resting right frontal active individuals should be higher in withdrawal attitude (Davidson & Fox, 1989). Because the pattern of frontal EEG asymmetry at rest was found to be stable across time (Schmidt, Cote, Santesso, & Milner, 2003; Tomarken, Davidson, Wheeler, & Kinney, 1992) and its appearance early in life is predictive of personality aspects (Fox et al., 2001), some have argued that this metric represents a “trait‐like” marker of dispositional affective style (Davidson, 1993, 2000).

In addition, a number of investigators have underlined the similarity of the BIS (Behavioral Inhibition System) and BAS (Behavioral Activation System) to the basic motivational constructs of approach and withdrawal (Carver, 1996; Fowles, 1988; Gray, 1994; Harmon‐Jones & Allen, 1997; Sutton & Davidson, 1997). Harmon‐Jones and Allen (1997) and Sutton and Davidson (1997) each examined the relationship between frontal EEG alpha asymmetry and these two constructs. In particular, the BAS should correspond to relatively greater trait left frontal brain activity. The BIS to the extent that it resembles the construct of withdrawal should correspond with to relatively greater right frontal brain activity. The neuroanatomical basis of the system is thought to be the left dorsolateral and medial prefrontal cortex and the basal ganglia. In contrast, the withdrawal system is activated by aversive conditions and leads to withdrawal behavior. The neuroanatomical basis of this system is supposed to be the right dorsolateral prefrontal cortex, the right temporal polar region, the amygdala, the basal ganglia, and the hypothalamus (Hewig et al., 2007). It was supposed that EEG asymmetry has trait components, and recent research underlines the relevance of this trait measure (BIS/BAS) to explain in deep this cortical asymmetry. Indeed, resting activity may elucidate the role of subjective attitude in responding to emotions, as a predictive marker of the left‐ or right‐asymmetry in specific emotion processing.

Nevertheless, it has to be noted that few studies have tried to connect emotional cue processing with the subjective attitude (BIS/BAS) and resting state brain activity, taking into account the impact of these factors on cortical system when it responds to specific emotional cues. Therefore, the first aim of the present research was to directly compare the contribution of personality component (BIS/BAS dichotomy). Moreover, the present study intended to explore the impact of resting activity for a successive lateralized response to emotional cue processing. Indeed, the resting state activity was considered as predictive of the subjective brain response to an emotional task, since this resting activity may be considered a predictive marker of the ability to respond to emotions (Raichle, 2010; Tortella‐Feliu et al., 2014), which has also been found to be associated with emotion regulation styles (e.g., Abler, Hofer, & Viviani, 2008; Berman et al., 2011; Bornas et al., 2013) and emotional cue comprehension (Balconi & Ferrari, 2012).

Moreover, it should be noted that no previous study has deeply explored the direct relationship between resting activity within the left and right PFC and the brain response to emotional cues using a multimethodological approach. Due to its fast temporal evolution and its representation among widespread cortical networks, both the resting state and the emotional cue processing should preferably be examined by means of methods that offer high resolution in both temporal and spatial domains. Among the different modalities available for monitoring brain activity and in addition to the EEG measure, near‐infrared spectroscopy (NIRS) is noninvasive and particularly well‐suited for evaluating the PFC activity, which we showed to be among the regions involved in emotional processing (Balconi, Grippa, & Vanutelli, 2015a). Interestingly, recent studies by NIRS investigating the neural correlates of emotion regulation processes also described an activation of the PFC (Balconi, Grippa, & Vanutelli, 2015b; Herrmann et al., 2008; Yang et al., 2007). For the reasons reported above, in addition to EEG, NIRS is particularly indicated to explore the brain activity in both resting state and active response to emotional stimuli. Moreover, NIRS and EEG measurement in a resting condition demonstrated that an increase of oxy‐hemoglobin (O2Hb) was associated with an increase in neuronal responsiveness, whereas a decrease in O2Hb was associated with a decrease in neuronal responsiveness (Butti et al., 2006; Hoshi, Kosaka, Xie, Kohri, & Tamura, 1998). Indeed, it was observed that spontaneous hemodynamic brain activity is not just random noise, but is specifically organized in the resting brain (for a review, see Fox & Raichle, 2007).

Based on these two measures, first we hypothesized that EEG asymmetry and NIRS‐measured O2Hb changes at rest in the PFC may explain asymmetry in PFC activity and predict the emotional response to an experimental condition in which subjects have to process emotional cues. Alpha band was used to explore the general brain activity as a function of resting/experimental condition, independently from the functional significance of the brain oscillations.

Second, we hypothesized that higher left activity at rest should be correlated with an increased left activity in experimental condition, whereas higher right activity should be correlated with an increased right activity in experimental condition. Subjective response to different stimulus category should be predicted by resting activity using both EEG and fNIRS. That is, we expected that a higher left resting activity (marked by both EEG and NIRS) will be paralleled by higher cortical responsiveness within the left hemisphere for positive stimuli. In contrast, a higher right resting activity will be paralleled by a higher responsiveness within the right hemisphere for negative stimuli.

Third, about the trait component, BIS/BAS measure may be a factor able to explain EEG and NIRS during resting and brain activity in response to emotional processing. Specifically we expected, from one hand, for the resting brain activity that a more left‐lateralized responsiveness should be supported by an increased BAS rating, whereas a more right‐lateralized activity should be supported by an increased BIS rating.

Finally, based on approach/withdrawal model of emotions, a significant and consistent higher left prefrontal brain response was supposed for approach emotional cues, whereas a consistent higher right prefrontal activation was expected in response to withdrawal emotional cues (Balconi & Mazza, 2010). That is, we expected that the significant role of BIS/BAS could affect the direct emotional processing, that is the prevalence in responding to approach emotional cues by high BAS and, conversely, to aversive cues by high BIS, with consistent related effects on EEG and hemodynamic profile within the two hemispheres: that is increased brain activity in response to approach emotions is expected for BAS within the left side, whereas an opposite trend is expected for BIS, with an enhanced activity for aversive stimuli within the right side.

## 2 Materials and Methods

### 2.1 Participants

In all, 21 subjects, 11 females and 10 males (*M* age = 27.65; *SD* = 4.99; range = 23–32), were participated in the experiment. Subjects were right‐handed, with normal or corrected‐to‐normal visual acuity. They were checked for their clinical profile using neuropsychological assessment and clinical interview: no psychopathological elements or psychiatric pathologies were observed for depression or anxiety construct (as indicated by Beck Depression Inventory II, total score ≤10, Beck, Steer, & Brown, 1996; for STAI, total score ≤39, Eysenck, Payne, & Derakshan, 2005). In addition, no relevant neurological pathologies were revealed for subjects or their family. Subjects gave informed written consent to participate in the study and the research was approved by the Ethical Committee institution where the work was carried out. The manuscript meets the ethical standards. The authors declare that they have no conflict of interests. All procedures performed in studies involving human participants were in accordance with the ethical standards of the institutional research committee and with the 1964 Helsinki declaration and its later amendments or comparable ethical standards. All the procedures were carried out with adequate understanding of the subjects, who read and signed the Research Consent Form. No payment was provided for their participation to the experiment.

### 2.2 Stimuli

Stimuli were taken from the International Affective Picture System (IAPS) (Bradley & Lang, 2007), depicting 40 pleasant and 40 unpleasant pictures, which were 20 low and 20 high arousing each, and 20 neutral stimuli. They were validated on valence and arousal ratings (Balconi, Brambilla, & Falbo, 2009). Indeed, the content groups were selected such that there was almost no overlap in affective valence ratings (pleasant, unpleasant, and neutral). Moreover, each emotional category included high arousing and low arousing (20 for each category) patterns. Neutral stimuli were rated as medium arousing in general. Thus, the image stimuli of each of the emotional categories varied in arousal (low to high) and valence (negative to positive). IAPS subjective ratings were obtained with the SAM (Self‐Assessment Manikin), using an easier adapted 5‐point version (Bradley & Lang, 1994, 2007). In a post‐experimental phase, subjects had time to rate their emotional experience on SAM evaluating valence and arousal on a bipolar scale applied to each picture (Bradley & Lang, 1994). Arousal and valence subjective ratings were analyzed with repeated measure ANOVAs (2 arousal × 2 valence). About the valence ratings, valence was significant (*F*_1,20_ = 8.08, *p *<* *.001): negative valenced stimuli were rated as more negative compared to positive stimuli. In parallel, about arousal ratings, arousal main effect was significant (*F*_1,20_ = 7.09, *p *<* *.001): low arousal stimuli were rated as lower on arousal compared to high arousal stimuli.

### 2.3 Procedure

**Figure 1:**
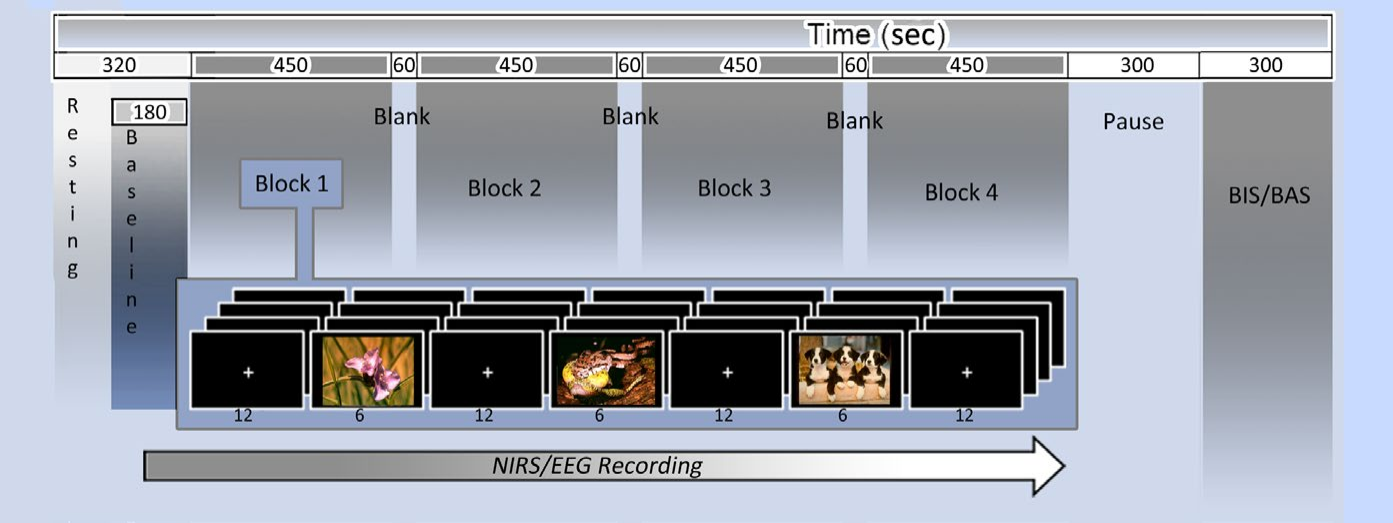
Experimental setting for electroencephalographic (EEG) and functional near‐infrared spectroscopy (fNIRS) recording

One hundred and eighty seconds baseline was registered at the beginning of the experiment before the picture series. This resting state time window was considered enough to support the successive comparison with the task period. In fact, this range is usually adopted in experimental setting (Fukuda et al., 2014; Medvedev, 2014). In addition, subjects were previously instructed to familiarize with the situation and the task before data acquisition and the effective resting state period was exclusively dedicated to relax their mind and remain motionless as much as possible and allow the mind to disengage during these periods (for this procedure, see Putman, van Peer, Maimari, & van der Werff, 2010). Participants performed resting eyes‐closed eye‐open baseline periods. The two modalities were counterbalanced across subjects. Because the eyes‐open and eyes‐closed resting states were considered to reflect baseline brain activity of different types, we included resting states of these two types in the present study (Allen & Cohen, 2010; Barry, Clarke, Johnstone, & Brown, 2009; Beaton et al., 2008; Marx et al., 2004; Nakao et al., 2013; Yan et al., 2009). Participants were seated in a dimly lit room, facing a computer monitor that was placed 70 cm from them. Stimuli were presented using STIM software (Stim^2^, Compumedics Neuroscan, Charlotte, North Carolina) running on a personal computer with a 15‐inch screen. Participants were required to observe each stimulus during fNIRS/EEG recording, and they should attend to the images the entire time of exposition. Pictures were shown in a random order in the center of a computer monitor for 6 s, with an inter‐stimulus interval of 12 s. A familiarization phase was foreseen, where subjects saw and rated five pictures, different from the stimuli used in the experimental phase (Figure 1).

### 2.4 BIS/BAS score

BIS and BAS scores were calculated for each subject (Carver & White, 1994; Leone, Pierro, & Mannetti, 2002). The evaluation included 24 items (20 score‐items and four fillers, each measured on four‐point Likert scale), and two total scores for BIS (range = 7–28; 7 items) and BAS (range = 13–52; 13 items). The questionnaire was submitted to the subject after the experimental phase. Based on these measures, two total scores (BIS and BAS total) were obtained. The mean and standard deviation values for each scale were, respectively, as follows: for BIS: 16.55(1.19); BAS: 36.40(1.23). Cronbach's alpha was calculated for BIS (0.88) and BAS (0.85). Since a preliminary analysis did not show any significant gender difference for BIS/BAS, we did not include this factor in the final category.

### 2.5 fNIRS

fNIRS measurements were conducted with the NIRScout System 88 (NIRx Medical Technologies, LLC. Los Angeles, California) RRID:SCR_014540 using a six‐channel array of optodes (four light sources/emitters and four detectors) covering the prefrontal area. Emitters were placed on positions AF3‐AF4 and F5‐F6, whereas detectors were placed on AFF1‐AFF2 and F3‐F4. For successive analyses, we considered the couples AF3/F3 and AF4/F4, resulting in channels AFF3/AFF4. Emitter–detector distance was 30 mm for contiguous optodes and near‐infrared light of two wavelengths (760 and 850 nm) was used. NIRS optodes were attached to the subject's head using a NIRS‐EEG compatible cup (international 10/5 system, Oostenveld & Praamstra, 2001).

With NIRStar Acquisition Software, changes in the concentration of oxy‐hemoglobin (O2Hb) and deoxy‐hemoglobin (HHb) were recorded from a 180 ms baseline before the stimulus presentation (the trial onset), using the modified Beer–Lambert law. To remove baseline drift and pulsation due to the heartbeat, the raw data of O2Hb and HHb from individual channels were digitally band‐pass filtered at 0.01–0.3 Hz. Motion artifacts were checked subject‐by‐subject both during the experimental phase and the signal analysis. Signals obtained from the six NIRS channels were measured with a sampling rate of 6.25 Hz. Then, they were analyzed and transformed according to their wavelength and location, resulting in values for the changes in the concentration of O2Hb and HHb hemoglobin for each channel. Hemoglobin quantity is scaled in mmol × mm, implying that all concentration changes depend on the path length of the NIR light in the brain. Then, the mean concentration of each channel within a subject was calculated by averaging data across the trials, from the trial onset for 6 s. In a preliminary analysis, both O2Hb and HHb were used. However, since no significant effect was found for HHB in that preliminary analysis, we reported only the O2Hb results at the final step.

To analyze the left/right asymmetry of PFC activity at rest, we calculated the Lateralized Index Response (LIR_nirs_, right AFF4‐left AFF3) for O2Hb (see also Ishikawa et al., 2014). The index provides values in the range of (−1, +1). A positive LIR_nirs_ shows that the right PFC is more active than the left PFC at rest, whereas a negative LIR_nirs_ indicates that the left PFC is more active than the right PFC at rest.

To determine the left/right asymmetry of PFC activity during the experimental task, we obtained a laterality index (LI_nirs_) for the O2Hb concentration changes (right AFF4‐left AFF3). LI_nirs_ > 0 indicates greater activity of the right PFC, whereas LI_nirs_ < 0 indicates greater activity of the left PFC. Applying Ishikawa's formula, we used ΔoxyRt (O2Hb concentration changes for right during the defined time period) and ΔoxyLt (O2Hb concentration changes for left during the defined time period, for the formula see Ishikawa et al., 2014) per se, instead of the variations from their minimum values, as reported in the original formula. This modification was adopted for both the numerator and denominator of the formula. It was done because we used ΔoxyRt and ΔoxyLt per se, instead of the variations from their minimum values. It allowed more “stable” values although in this case the denominator could be zero or near zero where the target quantity diverges.

### 2.6 EEG

A 16‐channel portable EEG‐System (V‐AMP: Brain Products, München, Germany) was used for EEG acquisition. A NIRS‐EEG compatible ElectroCap with Ag/AgCl electrodes was used to record EEG from active scalp sites referred to earlobe (10/5 system of electrode placement). EEG activity was recorded on the channels AFF3, AFF4, Fz, AFp1, AFp2, C3, C4, Cz, P3, P4, Pz, T7, T8, O1, and O2. The cap was fixed with a chin strap to prevent shifting during the task. The data were recorded during the resting period (see previous features for NIRS data acquisition) using a sampling rate of 500 Hz, with a notch filter of 50 Hz. The impedance of recording electrodes was monitored for each subject prior to data collection and it was always kept below 5 kΩ. Additionally, two EOG electrodes were sited on the outer canthi to detect eye movements. Blinks were also visually monitored. Ocular artifacts (including both eye movements and blinks) were corrected using an eye‐movement correction algorithm that applies a regression analysis in combination with artifact averaging (rejected epochs, 4%). After performing EOG correction and visual inspection, artifact‐free trials were used for the successive analysis. Artifact‐free epochs (1‐s) were analyzed using a discrete Fourier transform (DFT), with a Hanning window of one second width and 50% overlap. Power (microvolts squared) was derived from the DFT output in the alpha (8–13 Hz) frequency band. A natural log (ln) of EEG power was then performed for power at each site to reduce skewness. For the experimental phase, a time window of 1000 ms from the onset was used. To determine the left/right asymmetry of PFC activity during the resting/experimental period, we calculated a laterality index (respectively LIR_eeg_ for resting phase; LI_eeg_ for experimental phase) for the alpha changes. The alpha activity in the left side was subtracted from the alpha activity in the right side. The score was computed (log[left, AFF3‐log[right, AFF4]). Because alpha power has been found to be inversely related to the activation of the corresponding regions of the brain, anterior alpha asymmetry measurements could reflect relative differences in activity between the left and the right hemispheres (Blackhart, Kline, Donohue, La Rowe, & Joiner, 2002). That is, LIR_eeg_ and LI_eeg_ > 0 designates greater activity of the right PFC, whereas LIR_eeg_ and LI_eeg_ < 0 indicates greater activity of the left PFC. We opted to consider only the alpha band modulation within the entire band spectrum since we did not look for a “functional” analysis of the frequency bands. That is, we did not consider the low‐frequency bands (delta and theta) usually implicated in functional emotional processing. Instead, we opted to consider a generic brain activation index (alpha band modulation) which reflects the brain lateralization per se as cortical hemispheric response to the emotional stimuli.

### 2.7 sLORETA

To verify the alpha band source generators, a preliminary signal source localization was computed for spectral data to investigate intra‐cortical generators of electrophysiological alpha oscillation during resting and experimental condition. The standardized Low‐Resolution Brain Electromagnetic Tomography (sLORETA RRID:SCR_013829) was used to compute the cortical three‐dimensional distribution of current density. The description of the method is presented in Pascual‐Marqui (2002), and the proof of its exact, zero‐error localization property is described in Pascual‐Marqui (2007, 2009). Computations are made in a realistic head model (Fuchs, Kastner, Wagner, Hawes, & Ebersole, 2002), using the MNI152 template (Mazziotta et al., 2001), with the three‐dimensional solution space restricted to cortical gray matter, as determined by the probabilistic Talairach atlas (Lancaster et al., 2000). The intra‐cerebral volume is partitioned in 6239 voxels at 5 mm spatial resolution. We extracted sLORETA values (log‐transformed values) for alpha frequency band.

Signal source localization and contrast analyses were computed for spectral data to investigate intra‐cortical generators of electrophysiological oscillation. On the basis of estimated intra‐cortical distribution of current density and of significant contrast analyses between resting and experimental periods, we extracted sLORETA values (log‐transformed values) for alpha frequency band. Given that computation, greater differential indices mirror greater electrophysiological activation. Specifically, contrast analyses on current density data for alpha band were computed using a statistical non‐parametric mapping approach – SnPM – supplied by the sLORETA software (Nichols & Holmes, 2002). No variance smoothing factor has been applied to the computation. The significance threshold was set at *p *<* *.01 and based on a permutation test with 5000 permutations while errors due to multiple testing were controlled thanks to the SnPM methodology. We also applied a minimum cluster‐size threshold of 10 voxels. No preliminary normalization was performed on current density data. The statistical methodology is based on estimating, via randomization, the empirical probability distribution for the maximum of a *t*‐statistic on log‐transformed data under the null hypothesis. This computation corrects for multiple testing for all electrodes and, due to its non‐parametric nature, its validity need not rely on any assumption of Gaussianity.

### 2.8 Data analysis

A series of analysis was performed distinctly for the resting (a) and the experimental (b) phase on NIRS and EEG measures.

Specifically for the resting phase, to verify the effect of BIS/BAS on brain activity, two stepwise multiple regressions were applied, respectively, to LIR_eeg_‐ and LIR_nirs_‐dependent measures_._ Predictor variables were BIS and BAS ratings. Moreover, to test the direct relationship between LIR_eeg_ and LIR_nirs_, a successive Pearson correlational analysis was also applied to LIR_eeg_ and LIR_nirs_ measures. Due to multiple independent analyses and multiple comparisons, we successively applied Bonferroni test for inequality.

A second set of analyses was applied to experimental phase. A preliminary statistical comparison was made among the positive, negative, and neutral valence. Since neutral stimuli (as a comparative level) always differed, respectively, from positive and negative stimuli with lower values for both LI_eeg_ and LI_nirs_ (for all comparisons, *p *<* *.01), it was not included in the successive analysis, which used two levels‐two factors (valence and arousal). Therefore, successively to test a possible effect related to stimulus valence, two factor (2 arousal × 2 valence) repeated measure ANOVAs were applied distinctly to LI_eeg_ and LI_nirs_ dependent measures. Second, a set of regression analyses was performed to verify the effect of BIS/BAS and LIR_nirs_ as predictor on the LI_nirs_ measure as criterion. The same set of regression was performed for the EEG measures (similarly with BIS/BAS LIR_eeg_ as predictors and LI_eeg_ as criterion).

## 3 Results

### 3.1 Resting state phase

#### 3.1.1 Regression analysis

##### BIS/BAS and LIR_eeg_

**Figure 2:**
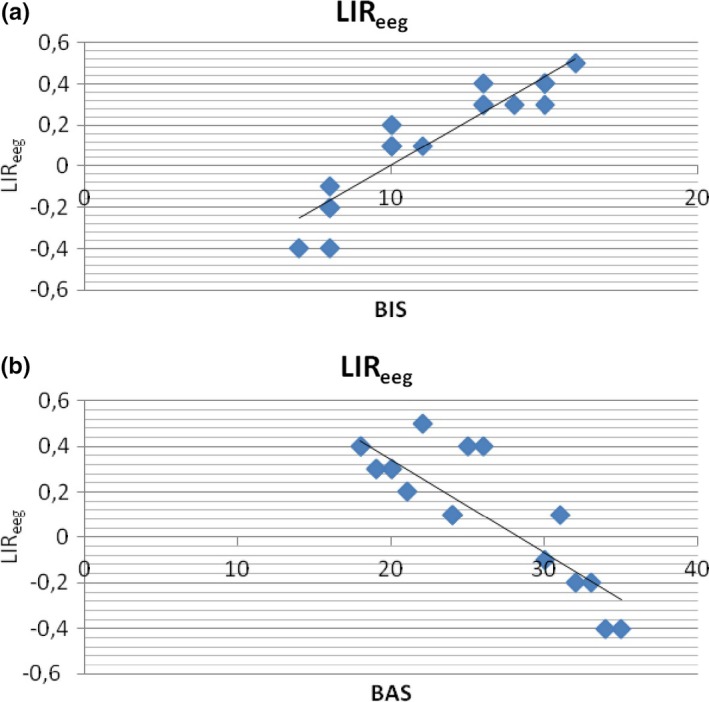
Stepwise Multiple Regression analysis. Behavioral Activation System (BAS), Behavioral Inhibition System (BIS) as predictor variables and lateralized Index Response (LIR_eeg_) as predicted variable

A stepwise multiple regression analysis was performed. Predictor variables were BIS/BAS scores and predicted variable was LIR_eeg_ modulation. In Table 1a,b, we report the cumulative multiple correlations between predictor and predicted variables (R), cumulative proportion of explained variance (*R* ^2^), and the regression weights (β) for the regression equation at each step of the multivariate analysis. As shown (Figure 2a,b), both BAS and BIS predicted the LIR_eeg_ variations. Indeed, increased LIR_eeg_ (more right activity) was related to increased BIS rating, whereas decreased LIR_eeg_ (more left activity) was related to increased BAS rating.

**Table 1:** Stepwise multiple regressions. (a) BAS and BIS as predictor variables and LIR_eeg_ as predicted variable. (b) BAS and BIS as predictor variables, LIR_nirs_ as predicted variable

Predictor	BAS	BIS
Model	1	2
(a)		
LIR_eeg_		
*R*	0.51	0.96
*R* ^2^	0.26	0.92
Β	0.34	0.28
*SE*	0.18	0.27
*T*	**2.09** *	**1.97** *
(b)		
LIR_nirs_		
*R*	0.42	0.89
*R* ^2^	0.17	0.79
Β	0.31	0.32
*SE*	0.25	0.20
*T*	**1.87** *	**1.95** *

BAS, Behavioral Activation System; BIS, Behavioral Inhibition System; LIR_eeg_ and LIR_nirs,_ Lateralized Index Response.

* = ≤ .01. Bold values are significant effect.

##### BIS/BAS and LIR_nirs_

**Figure 3:**
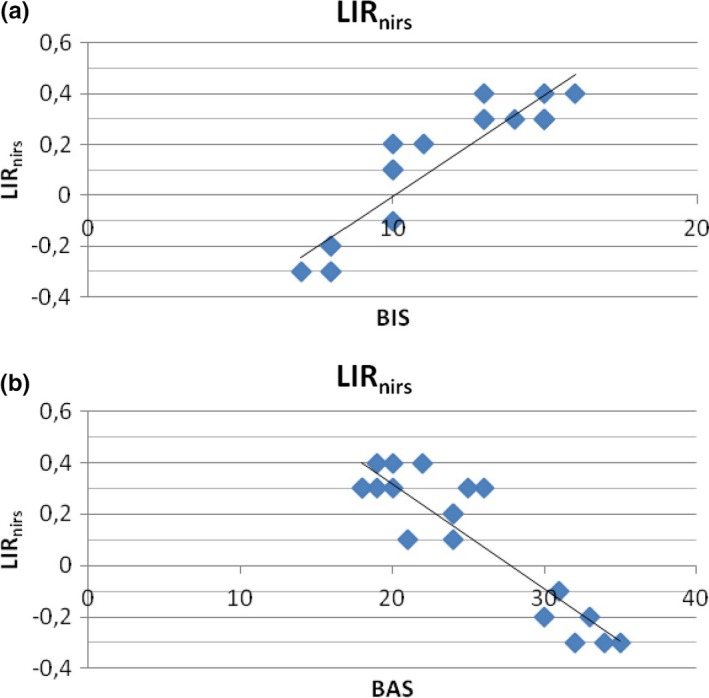
Stepwise Multiple Regression analysis. Behavioral Activation System (BAS), Behavioral Inhibition System (BIS) as predictor variables and lateralized Index Response (LIR_nirs_) as predicted variable

The second stepwise regression analysis was applied to LIR_nirs_. Also in this case BIS/BAS construct predicted the LIR_nirs_ modulation (Figure 3a,b). That is, increased LIR_nirs_ was explained by increased BIS values, whereas decreased LIR_nirs_ was explained by increased BAS values.

#### 3.1.2 Correlational analysis between LIR_nirs_ /LIR_eeg_

Pearson correlational analysis was applied to LIR_eeg_ and LIR_nirs_ in resting condition. Significant Pearson correlational values were found between LIR_eeg_ and LIR_nirs_ (*r *=* *.593; *p *<* *.01). Indeed, a positive correlation was found between LI_eeg_ and increased LI_nirs_. That is, the increasing positive values (more right activity) or decreasing negative values (more left activity) were consonant between the electrophysiological and hemodynamic measures.

### 3.2 Experimental phase

**Figure 4:**
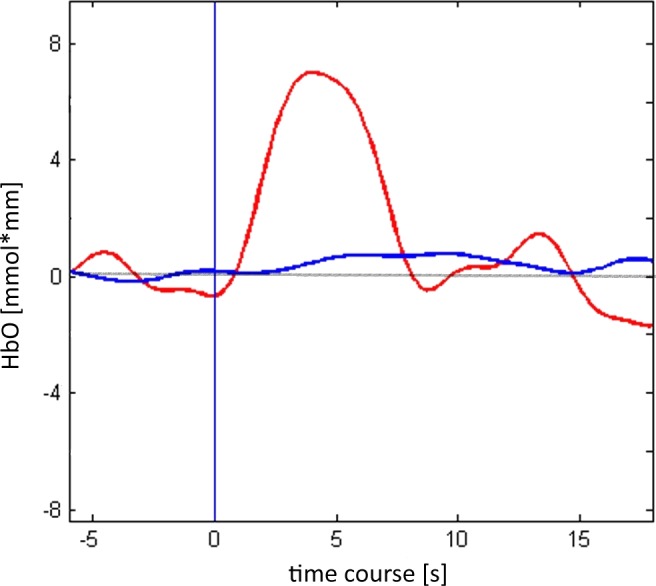
Temporal course display of 02Hb (red), and HHb (blu). 0 represent the beginning of the experimental period. Mean values AFF3/AFF4 reported

The cerebral blood oxygenation changes in the bilateral PFC were monitored by NIRS also during the experimental phase. The first 320‐s period before the task was used as baseline and was subtracted from the mean activation values (measured throughout task performance). The time course of O2Hb, HHb, and Total Hb changes during the experimental condition showed, respectively, a peak at about 4.8, 5.3, and 5.2 s after the stimulus onset. During the inter‐stimulus interval, we had reduced changes for O2Hb, HHb, and Total Hb, with a modulation, respectively, at about 5–6 s from the beginning of the inter‐stimulus interval (Figure 4).

#### 3.2.1 ANOVA

A repeated measure ANOVAs (independent factors: 2 arousal × 2 valence) were applied distinctly to LI_eeg_ and LI_nirs_ dependent measures. For all of the ANOVA tests, degrees of freedom were corrected by Greenhouse‐Geisser epsilon when appropriate. Contrast analyses (paired comparisons) were applied to significant main or interactions effects.

##### LI_eeg_ (a)

**Figure 5:**
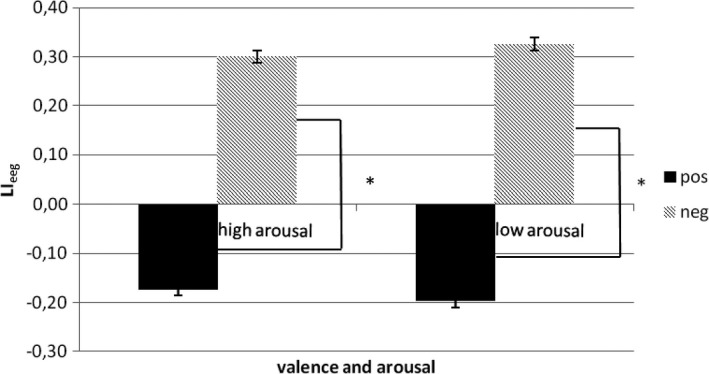
LI_eeg_ as a function of valence and arousal. Significant differences were found between positive and negative cues

As shown by ANOVA, a main effect of valence (*F*_1,20_ = 9.11, *p* < .001) was significant at the analysis. Indeed, LI_eeg_ values were higher (more right activity) for negative stimuli, and lower (more left activity) for positive stimuli. Arousal *F*_1,20_ = 0.87, *p* = .60 main effects, and valence × arousal (*F*_1,20_ = 1.37, *p* = .45) interaction effects were not significant (Figure 5).

##### LI_nirs_ (b)

**Figure 6:**
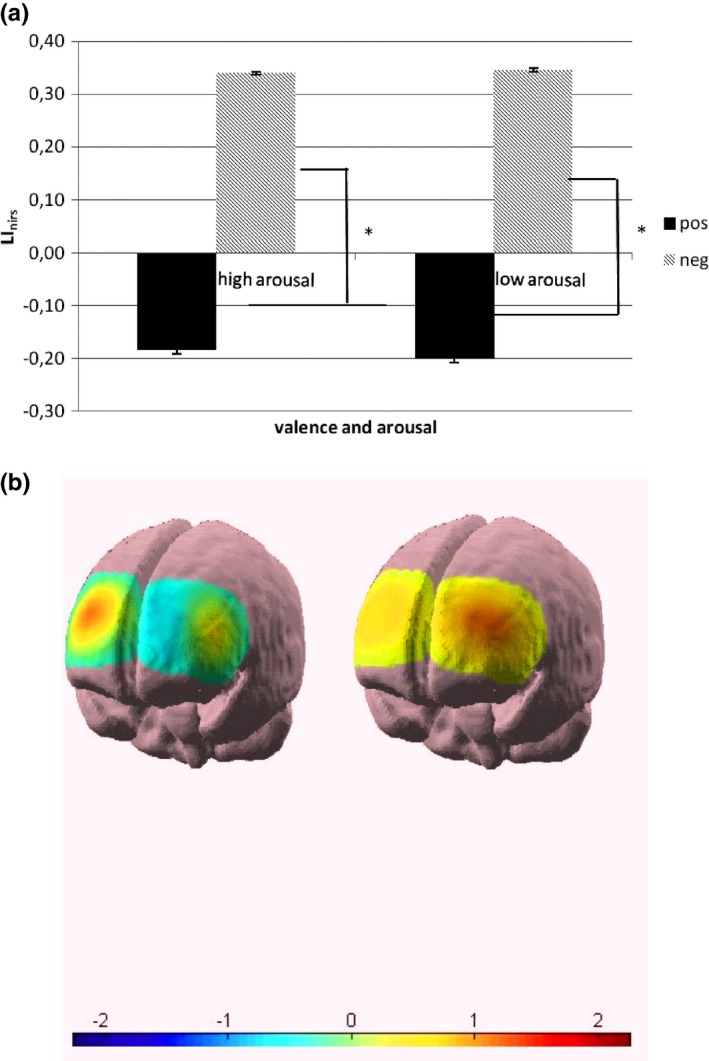
(a) LI_nirs_ as a function of valence and arousal. Significant differences were found between positive and negative cues. (b) Cortical maps (three channels represented, mean response during stimuli presentation). The concentration of O2Hb. LI_nirs_ values were positive (more right activity) in response to negative stimuli (left image), and negative (more left activity) in response to positive stimuli (right image)

As shown by ANOVA, the main effect of valence (*F*_1,20_ = 12.09, *p* < .001) was significant at the analysis. Indeed, LI_nirs_ values were higher (positive values, more right activity) for negative stimuli, and lower (negative values, more left activity) for positive stimuli. In contrast, arousal (*F*_1,20_ = 0.80, *p* = .57) and valence × arousal (*F*_1,20_ = 1.03, *p* = .39) were not significant (Figure 6a,b). In the topographical maps, three channels were represented for each hemisphere, and the figure reported the mean response during positive and negative stimuli presentation.

#### 3.2.2 sLORETA source analysis

**Figure 7:**
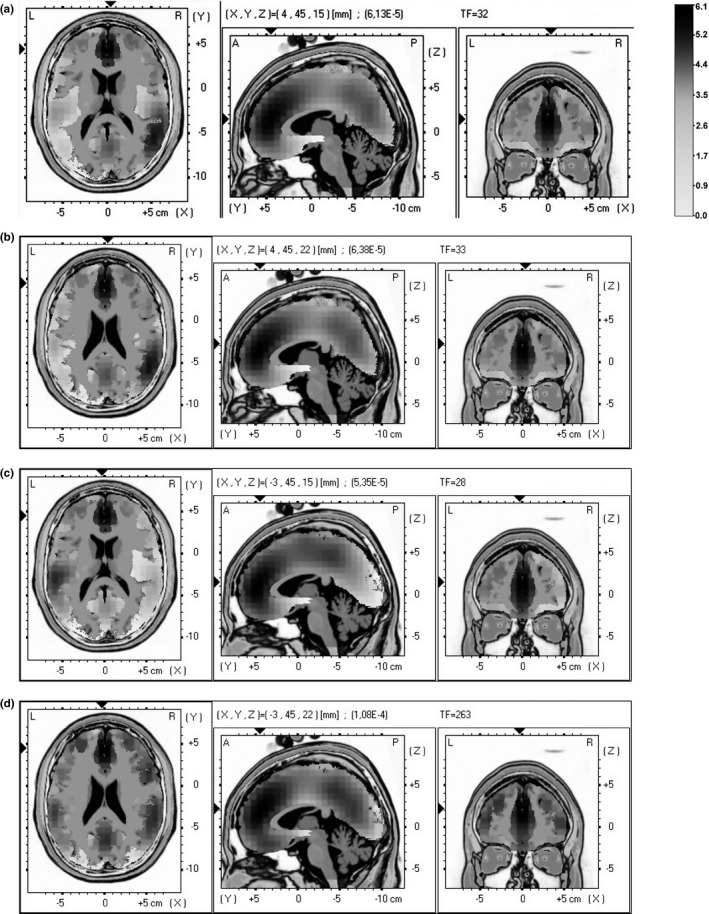
Results of the sLORETA analysis. The image shows the LORETA slices in Talairach space for the estimated source of activation differences comparing resting with experimental condition for positive and negative stimuli (all subjects)

To estimate the localization of the source of the cortical differences for resting and experimental condition, sLORETA was performed comparing the two conditions for each type of stimuli (positive and negative). No significant differences were revealed for resting and experimental condition in response to positive/negative stimuli. The algorithm localized the source of this activation to Talairach BA9 and BA10 (for positive stimuli: *t* = 1.03, *p* = .43; for resting *x* = 4, *y* = 45, *z* = 15; for experimental *x* = 4, *y* = 45, *z* = 22; for negative stimuli: *t* = 1.18, *p* = .35; for resting *x* = −3, *y* = 45, *z* = 15; for experimental *x* = −3, *y* = 45, *z* = 22) (Figure 7a–d).

#### 3.2.3 Regression analysis between BIS/BAS‐LIR_eeg_ and LI_eeg_

Distinct stepwise multiple regression analyses were performed. Predictor variables were BIS/BAS scores and LIR_eeg_ measure and the predicted variable was LI_eeg_ modulation in response, respectively, to positive and negative cues. In Table 2a, we report the cumulative multiple correlations between predictor and predicted variables (*R*), cumulative proportion of explained variance (*R* ^2^), and the regression weights (β) for the regression equation at each step of the multivariate analysis.

**Table 2:** Stepwise multiple regressions. (a) BAS, BIS, and LIR_eeg_ as predictor variables, LI_eeg_ as predicted variable. (b) BAS, BIS, and LIR_nirs_ as predictor variables, LI_nirs_ as predicted variable

Predictor	Positive			Negative		
	BAS	BIS	LIR_eeg_	BAS	BIS	LIR_eeg_
Model	1	2	3	1	2	3
(a)						
LI_eeg_						
*R*	0.47	0.58	0.91	0.18	0.60	0.80
*R* ^2^	0.22	0.33	0.82	0.04	0.36	0.64
Β	0.34	0.17	0.27	0.20	0.23	0.15
*SE*	0.18	0.20	0.32	0.15	0.20	0.26
*T*	**1.92** *	1.54*	**1.63** *	0.95	**1.87** *	0.78

Predictor	Positive			Negative		
	BAS	BIS	LIR_nirs_	BAS	BIS	LIR_nirs_
Model	1	2	3	1	2	3
(b)						
LI_nirs_						
*R*	0.49	0.64	0.97	0.21	0.59	0.95
*R* ^2^	0.24	0.40	0.87	0.04	0.34	0.81
Β	0.29	0.22	0.31	0.23	0.21	0.27
*SE*	0.25	0.20	0.26	0.20	0.25	0.17
*T*	**1.98** *	1.03	**1.78** *	0.85	**1.85** *	**1.73** *

BAS, Behavioral Activation System; BIS, Behavioral Inhibition System; LIR_eeg_ and LIR_nirs,_ Lateralized Index Response.

* = ≤ .01. Bold values are significant effect.

**Figure 8:**
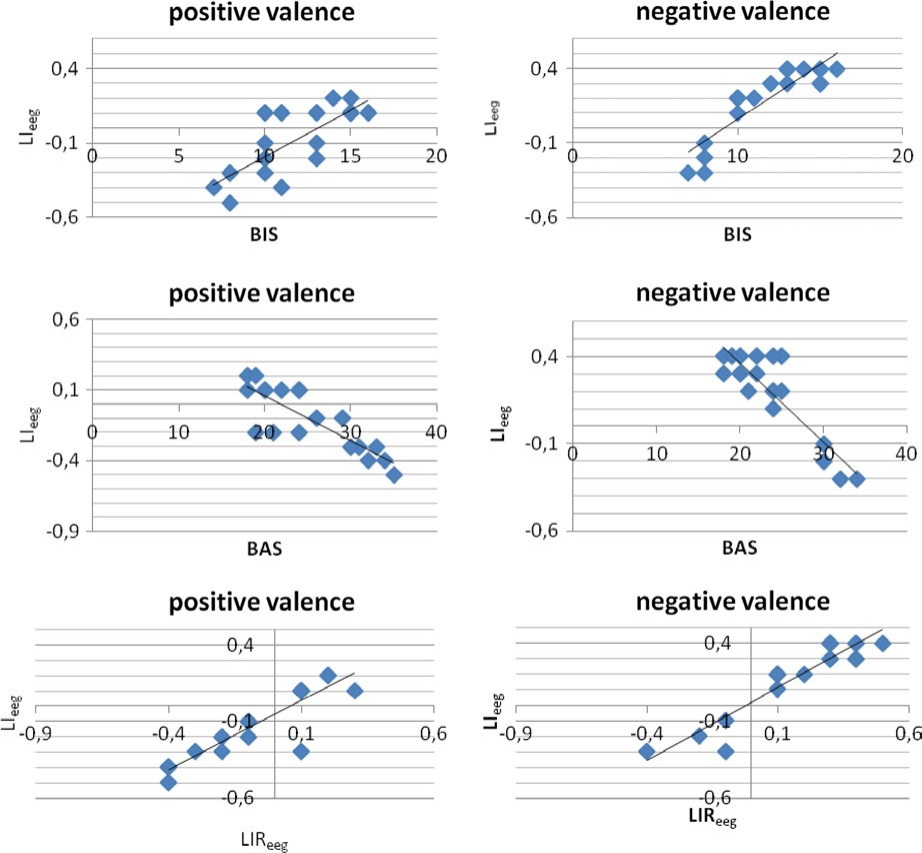
Stepwise Multiple Regression analyses (left: for positive cues; right for negative cues). Behavioral Activation System (BAS), Behavioral Inhibition System (BIS) and lateralized Index Response (LIR_eeg_) as predictor variables, LI_eeg_ as predicted variable

As shown, BAS score and LIR_eeg_ modulation predicted the LI_eeg_ value in response to positive stimuli. Indeed, an increased BAS and decreased LIR_eeg_ (more left brain activity) predict LI_eeg_ decreasing (more left activity). In addition, BIS score predicted the LI_eeg_ value in response to negative stimuli: in fact increased BIS values predict increased LI_eeg_ (more right activity) (Figure 8).

#### 3.2.4 Regression analysis between BIS/BAS LIR_nirs_ and LI_nirs_

Distinct stepwise multiple regression analyses were performed. Predictor variables were BIS/BAS score and LIR_nirs_, and the predicted variables was LI_nirs_ modulation in response to positive and negative cues (Table 2b).

**Figure 9:**
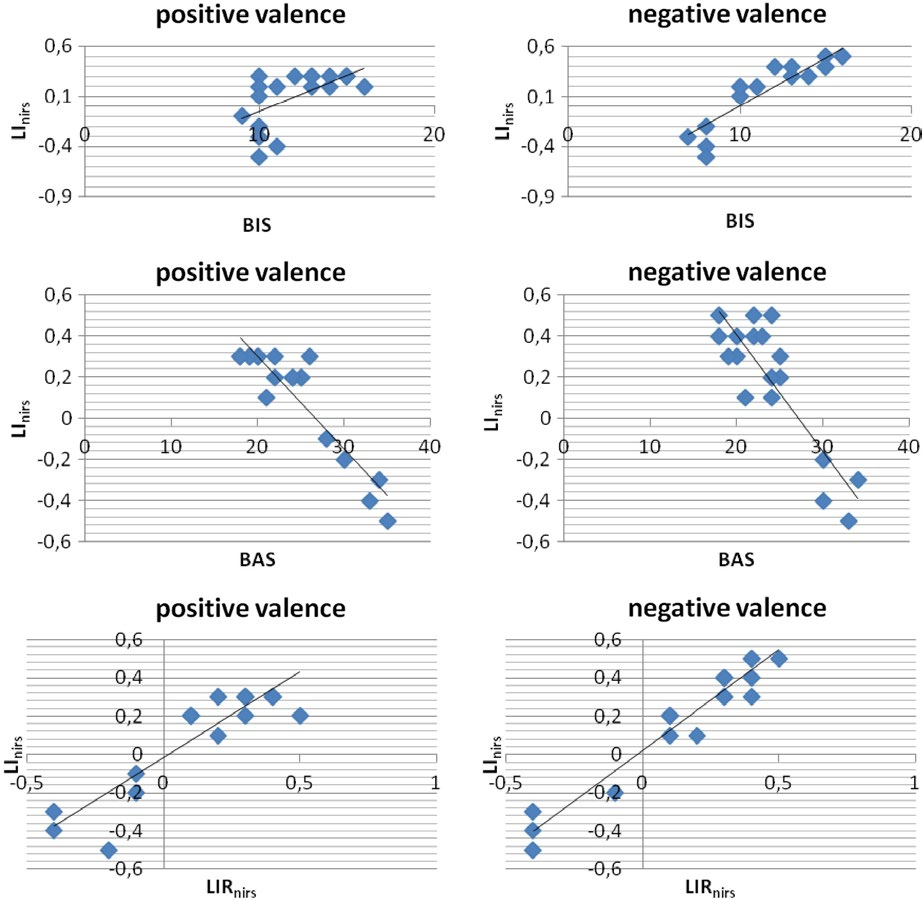
Stepwise Multiple Regression analyses (left: for positive cues; right for negative cues). Behavioral Activation System (BAS), Behavioral Inhibition System (BIS) and lateralized Index Response (LIR_nirs_ ) as predictor variables, LI_nirs_ as predicted variable

As shown, BAS score and LIR_nirs_ modulation predicted the LI_nirs_ in response to positive stimuli. Indeed, an increased BAS and decreased LIR_nirs_ (more left brain activity) predict LI_nirs_ decreasing (more left activity). In contrast, BIS and LIR_nirs_ modulation predicted the LI_nirs_ in response to negative stimuli: decreased BAS and increased LIR_nirs_ (more right brain activity) predict LI_nirs_ increasing (more right activity) (Figure 9). No other effect was statistically significant.

#### 3.2.5 Correlational analysis between LI_eeg_ and LI_nirs_

Pearson correlational analysis was applied to LI_eeg_ and LI_nirs_. Significant Pearson correlational values were found between LI_eeg_ and LI_nirs_. That is, a significant correlation was found between increased LI_eeg_ and increased LI_nirs_ (more right activity) in response to negative stimuli (*r *=* *.597; *p *<* *.01). Moreover, a positive correlation was found between decreased LI_eeg_ and decreased LI_nirs_ (more left activity) in response to positive stimuli (*r *=* *.583; *p *<* *.01).

## 4 Discussion

The present paper aimed to explore the direct relationship between the BIS/BAS trait component, lateralized resting brain activity within PFC, and brain response to emotional cues as a function of stimulus valence. First, we found that the lateralization of alpha brain oscillations (EEG) and of hemodynamic measures (fNIRS) in resting state affected the successive lateralized brain response to emotions. A second result was the specificity of this lateralization effect in response to emotions with respect of valence. Indeed, we observed the significant differences between positive and negative cues related, respectively, to the left and right hemisphere activation. Moreover, BIS/BAS components were found to predict both the resting state and the successive lateralized brain response to emotional cues. Therefore, BIS/BAS was interpreted as a predictive trait measure able to explain the resting and brain activity modulation. Finally, a significant correlation was systematically found between EEG and hemodynamic measures.

The first main result of the present research was the relationship between the lateralized prefrontal activity at rest and the lateralized brain response to emotional stimuli for both EEG and fNIRS measures. Indeed, as indicated by regression analysis results, the resting state highly predicts the subjects’ responses to the emotional cues. We found that subjects with more right‐dominant activity at rest (positive values of LIR_eeg_ and LIR_nirs_) showed higher right activity during the emotional task (positive LI_eeg_ and LI_nirs_ scores), while those with left‐dominant changes at rest (negative values of LIR_eeg_ and LIR_nirs_) showed higher left activity (negative values for LI_eeg_ and LI_nirs_). Due to the consonance of these two different measures, we can suppose that the resting state predicted the successive EEG/hemodynamic activity in the brain. The source localization confirmed that the direct relationship between the EEG/fNIRS measures implicated the same brain activity. Indeed, the brain correlations (BA9) found by LORETA were resembled by the PFC activation revealed by the hemodynamic measure.

However, the second main result of the present research was that this “predictive effect” was not independent from the stimulus valence, since it was observed in strength relationship with the emotional content of the stimuli. Indeed, we observed that more right activity during the resting state explained the successive increased right hemisphere only in response to negative cues, and that more left activity during rest supported the more left activity only in response to positive cues. That is, subjects with more left or right dominant PFC activity at rest also exhibit concomitantly more left or right dominant PFC activity during the experimental condition in response, respectively, to the positive versus negative value of the emotional stimuli.

To synthesize, the predictive role of resting brain activity for the successive brain activation in response to the emotional stimuli was specifically related to the stimulus valence. The PCF clearly supported this mechanism of hemispheric lateralization. Indeed, the PFC was found to be the brain area able to explain this valence‐related emotional cue processing. This result was similar to those of previous research which found that the PFC plays a relevant role in the integration of different aspects of memory, and emotional regulation by orienting the cognitive control over emotional stimuli and more generally over emotional behavior (Balconi & Ferrari, 2012; Hariri, Bookheimer, & Mazziotta, 2000; Kalish & Robins, 2006; Knight, Staines, Swick, & Chao, 1999; Miller & Cohen, 2001). Specifically, more recent studies have identified the PFC as a key region in the experience and regulation of emotional responses (Balconi & Bortolotti, 2012; Balconi, Bortolotti, & Gonzaga, 2011; Damasio, 1996; Davidson, 2002; Ochsner & Gross, 2005). Similar effects were also found by previous studies which used classical neuroimaging measures. In an exploratory fMRI study with healthy subjects, it has been demonstrated that measures of emotions correlate with induced activity during rest within regions including the PFC components (Wiebking et al., 2011). However, these results were not related to other measures (such as brain oscillations) and they did not accurately monitored the time modulation of resting/task response due to the limitation of fMRI acquisition. Moreover, in many cases, fMRI studies compared specific clinical sample activity to healthy normal subjects in resting state, or they specifically focused on generic connectivity issue (Zhou, Yao, Fairchild, Zhang, & Wang, 2015).

For the reasons reported above, we might suggest that the clear lateralization effect observed in both resting and experimental conditions confirmed what was predicted by the valence model. However, instead of positive/negative valence distinction, other research suggested a dichotomy on approach/avoidance attitude to emotions, being the first more left‐hemisphere supported and the second more right‐hemisphere supported (Balconi & Mazza, 2009; Davidson, 1995; Harmon‐Jones, 2003). To explain these results, we can consider Davidson's model, which specifies that high levels of relative left frontal activity are related to the expression and experience of positive, approach‐related emotional conditions, and high levels of relative right frontal activity are related to the experience and expression of negative, withdrawal‐related emotions. In contrast, Harmon‐Jones (2004) has supposed that we may integrate these two approaches to include both motivational and valence components. Through the development of these competing hypotheses, Harmon‐Jones, Vaughn‐Scott, Mohr, Sigelman, and Harmon‐Jones (2004) have pursued the goal of determining more precisely what the emotional and motivational functions of asymmetrical frontal brain responsiveness might be. In doing so, they have specified a valence model of brain asymmetry in which high levels of relative left frontal activity are directly related to the expression and experience of positive emotions and high levels of relative right frontal activity are directly related with the experience and expression of negative emotions. Second, they have introduced motivational direction model in which high levels of relative left frontal activity are related to the expression of approach‐related emotions and high levels of relative right frontal activity are related to the expression of withdrawal‐related emotions. Although positive emotions are typically associated with attitude to approach and negative emotions are typically associated with attitude to withdraw, there are significant exceptions (e.g., anger). Therefore, future research should more directly compare different emotional patterns (e.g., comparing anger and fear) to better explain the contribution of valence and approach/avoidance models in supporting the lateralization effect.

The third main result was related to the significance that BIS/BAS dichotomy had for the EEG/fNIRS measure. Indeed, higher BAS score versus higher BIS score showed a significant impact on the resting state. As supposed by the approach/avoidance model, the lateralized response may be associated to these two distinct attitudes (Gray, 1990). However, also contrasting results were found (Hewig et al., 2007). In the present research, a significant and consistent relationship between the attitude model and the brain response was found not only during the resting state but contemporarily also during the emotional cue processing. In fact, as shown by regression analyses, BAS mainly explained the consistent modulation of the lateralized left brain activity during the resting state and the successive left brain response mainly to positive stimulus processing, whereas BIS mainly explained the modulation of the right brain activity at rest and the successive right hemisphere lateralization mainly in response to negative stimuli.

Based on the present results, we may suppose that the BIS/BAS trait effect on resting state may explain the role of possible stable subjective components in brain lateralization. Indeed, the results of the present research are consistent with the valence asymmetry model which supposes that the left/right asymmetry of PFC activity is correlated with specific emotional personality traits (Canli et al., 2001; Davidson, Jackson, & Kalin, 2000; Fischer, Andersson, Furmark, Wik, & Fredrikson, 2002). That is, the main effect of this “lateralization,” as shown by resting brain activity, is that each subject shows a specific “attitude,” and that this trait is observable in both a relative main left or right hemispheric activation also in the absence of specific emotional task. According to the asymmetry model, the left/right asymmetry of the PFC activity is correlated with specific emotional responses to stimuli (Canli et al., 2001; Davidson et al., 2000; Fischer et al., 2002). Indeed, some EEG and neuroimaging studies have revealed that people who show greater relative left PFC activity exhibit more positive and less negative dispositional mood (Tomarken et al., 1992) than their right‐dominant counterparts. In contrast, right frontally activated subjects respond more to negative affective challenges and less to positive affective challenges than their left dominant counterparts (Wheeler, Davidson, & Tomarken, 1993). However, it should be noted that, since in the present study, we used a compound index (i.e., left or right higher brain activity as a function of the contralateral brain activity) and not an absolute right versus left hemisphere activation, the results we obtained might be considered as a measure which expresses the “balanced” activity between left or right brain activity and not lateralization in terms of absolute left/right prevalent brain activity. For these reasons, future research should evaluate more deeply the independent effect of the left versus right hemisphere considering both resting and experimental conditions.

Nevertheless, results suggested a significant lateralized index mainly explained by BIS/BAS within PFC also during a specific emotional task, based on the positive (more directly left‐hemisphere balanced) and the negative (more directly right‐hemisphere balanced) valence of emotions (Balconi & Mazza, 2010; Everhart et al., 2003). Therefore, we may suppose that this personal attitude is able to orient the brain responsiveness not only during resting state, but also during emotional cue processing. Indeed, regression analysis revealed a significant predictive role of BIS/BAS measure in explaining the lateralized response to positive versus negative emotions. The role temperament and personality play in influencing emotional responses was confirmed by previous empirical research, for both normal and pathological samples (Everhart & Harrison, 2000; Heller, 1993; Mardaga, Laloyaux, & Hansenne, 2006). It was suggested that the bases of the emotional motivations correspond to two general systems for regulating adaptive behavior (Carver & White, 1994; Gray, 1981). The first system has withdrawal functions to processing potential threat cues, referred to as BIS (Gray, 1990; Lang, Bradley, & Cuthbert, 1990). A second system supports the engagement of action, referred to as BAS (Fowles, 1980). In clinical conditions, “unbalance effects” between left versus right activity may predict some pathological conditions, as shown in case of anxiety disturbs. Indeed, it was found that higher level of anxiety might be due to dysfunctional increased activity of the frontal right hemisphere or to a reduced activity of frontal left hemisphere (Van Honk et al., 1999; Zwanzger, Fallgatter, Zavorotnyy, & Padberg, 2009).

Finally, based on the correlational analysis, a relevant result was related to the similarity of the profiles revealed for brain oscillations and hemodynamic measure. Both NIRS and EEG measures showed a strength relationship each other. The EEG‐NIRS analysis showed significant linear associations between the O2Hb modulation and brain oscillation (alpha) value. This result was confirmed by the overlapping of the cortical generators (sLORETA) and the brain areas more responsive to emotional condition as shown by fNIRS. In NIRS studies, modulation of O2Hb during activation implies evoked changes of rCBF in response to neuronal activation, since changes in O2Hb are correlated with changes in rCBF. In addition, simultaneous measurements of NIRS and EEG at rest demonstrated a relationship between O2Hb change and mean EEG peak frequency (Hoshi et al., 1998). These results may suggest that changes of O2Hb concentration at rest measured by NIRS reflect neuronal activity at rest, as well as EEG resting activity.

The simultaneous recording of EEG and NIRS was previously indicated as an useful measurement for emotional studies (Doi, Nishitani, & Shinohara, 2013). More specifically, the use of NIRS to measure response to emotional cues in a topographic approach allows to analyze regional brain activity changes that are due to emotional manipulations in general, and to compare certain EEG effects to the regional hemodynamic modulation (Herrmann et al., 2008). This joined measure enables us to test whether and how specific emotion‐related EEG changes are related to distinct cortical activation patterns within the network which supports emotion perception (Schneider et al., 2014). Moreover, applied to resting state and to emotional cue processing, it may better elucidate the significance that cortical EEG and hemodynamic modifications have for distinct brain activities, the first based on resting condition and the second as an active response to external stimuli.

However, it should be considered that in the present research we opted to analyze only the high‐frequency alpha band modulation since we were not directly interested in the “functional” significance of EEG spectrum. That is, we considered the brain lateralization effect based on the typical frequency band responsive to the cortical activity (alpha band modulation) which may reflect the brain lateralization per se without a specific functional values. A complete analysis which may include the entire spectrum should furnish integrative and “functional” information on the role of the frontal and the other (not specifically frontal) brain areas.

## 5 Conclusion

To summarize, BIS/BAS measures showed their significance to explain the resting activity and brain responsiveness to emotions. That is, this trait component was able to support the individual differences in the resting state and to predict the emotional behavior during the task as a function of stimulus valence. The trait component may explain the lateralization effect and emotional behavior, more approach‐related for positive cues; more avoidance‐related for negative cues. These results were coherently supported by both oscillation and hemodynamic modulation. However, future research should better explain the reciprocal relationship between EEG and NIRS, taking into account the specific role they may have for emotions. Moreover, an exhaustive analysis of other cortical site contribution, in addition to the PFC, may better describe the role of other brain areas in emotion processing, to define the cortical map underlying this mechanism. Finally, the distinction between BAS and BIS component should be considered in depth in successive research. Indeed, their relationship with frontal asymmetry in resting state and in response to emotional cue should be better explored, taking into account BAS/BIS relationship with, respectively, approach/avoidance model and negative/positive valence model.

## Conflicts of Interest

None declared.
